# Natal Teeth: A Case Report and Reappraisal

**DOI:** 10.1155/2015/147580

**Published:** 2015-02-04

**Authors:** Ghadah A. Malki, Emad A. Al-Badawi, Mohammad A. Dahlan

**Affiliations:** North Jeddah Specialty Dental Center, Jeddah 23532, Saudi Arabia

## Abstract

The presence of teeth at birth (natal teeth) or within a month after delivery (neonatal teeth) is a rare condition. Natal and neonatal teeth are conditions of significant importance to pediatric dentists and pediatricians. This report discusses a case in which a five-day-old infant required extraction of a mobile mandibular natal tooth to avoid the risk of aspiration and interference with feeding. Also, a review of the literature was conducted to discuss the etiology, clinical features, complications, and management of natal and neonatal teeth.

## 1. Introduction

Normal eruption of primary teeth begins with the eruption of mandibular incisors at around 6 months of age [1]. Prematurely erupted primary teeth are referred to as congenital teeth, predeciduous teeth, fetal teeth, and dentitia praecox [2, 3]. Massler and Savaral [4] defined tooth/teeth present at birth as “natal teeth” and those erupting during the first month of life as “neonatal teeth.”

The difference between “early eruption” and “premature eruption” of natal and neonatal teeth is that “early eruption” occurs due to endocrine system changes while “premature eruption” is considered a pathological phenomenon as incomplete root formation causing the tooth to exfoliate in a short time period [5].

Natal and neonatal teeth are commonly present in the mandibular incisor region with a 66% predilection for females [6]. The prevalence of natal teeth has been investigated by several studies and different ranges have been reported from 1 : 716 to 1 : 3500 live births [3, 6–8].

Most of natal and neonatal teeth are considered early erupting teeth of the normal deciduous dentition [6] and the reported incidence of supernumerary teeth ranges from 1 to 10%.

The exact etiology is not known. Several sources suggest a possible hereditary component [4, 6, 9, 10]. An autosomal dominant gene was suggested which was substantiated by a report of a family of 5 siblings who were born with natal teeth [8, 11]. In a study that was conducted on Alaskan Tlingit Indians, the prevalence of natal or neonatal teeth was 9% of their newborns; interestingly enough 62% of the newborns relatives were also affected [10]. Furthermore, a positive family history of 7 out of 38 cases of natal and neonatal teeth was found by Kates et al [6].

Environmental factors, particularly polychlorinated biphenyls, appear to increase the incidence of natal teeth [12–15]. These exposed children usually display other accompanying symptoms, such as dystrophic fingernails and hyperpigmentation.

Natal teeth have been associated with a number of developmental abnormalities and various syndromes, including cleft lip and palate, Pfeiffer, Ellis-van Creveld (chondroectodermal dysplasia), Rubinstein-Taybi, steatocystoma multiplex, pachyonychia congenita (Jadassohn-Lewandowsky), cyclopia, Hallermann-Streiff (Mandibulo-oculo-facial dyscephaly with hypotrichosis), Pierre-Robin, Wiedeman-Rautenstrauch (neonatal progeria), Pallister-Hall, ectodermal dysplasia, craniofacial dysostosis, multiple adrenogenital, Sotos, steatocystoma, epidermolysis bullosa simplex, and Walker-Warburg syndrome [1, 7, 16–24].

It has been proposed that early erupting primary teeth could be due to abnormal location of the developing tooth germ in relation to the alveolar bone [25]. It was also suggested that this could be the result of hereditary influences [9]. However, in most cases the pathogenetic factors are impossible to identify. Therefore, careful evaluation of these infants is highly recommended.

Natal teeth management is dependent on a number of factors. If the natal tooth is supernumerary, then the treatment of choice is extraction. When the tooth/teeth are excessively mobile, extraction is indicated owing to the risk of exfoliation and swallowing or aspiration. However, when reviewing the literature, no reported cases of aspiration of natal or neonatal teeth were found. In one study, only 38% of natal and neonatal teeth exfoliated in the first year of life [26]. When natal teeth are only slightly mobile, they often stabilize soon after eruption. The most common complaint of natal and neonatal teeth was found to be trauma to the tongue on the tip or ventral surface, a complication referred to as Riga-Fede syndrome [4]. It occurs in 6–10% of cases of natal teeth [27, 28]. It was also suggested that this ulceration could be due to the fact that the tongue in infants lies immediately between the alveolar ridges [4, 29].

## 2. Case Report

**Figure 1:**
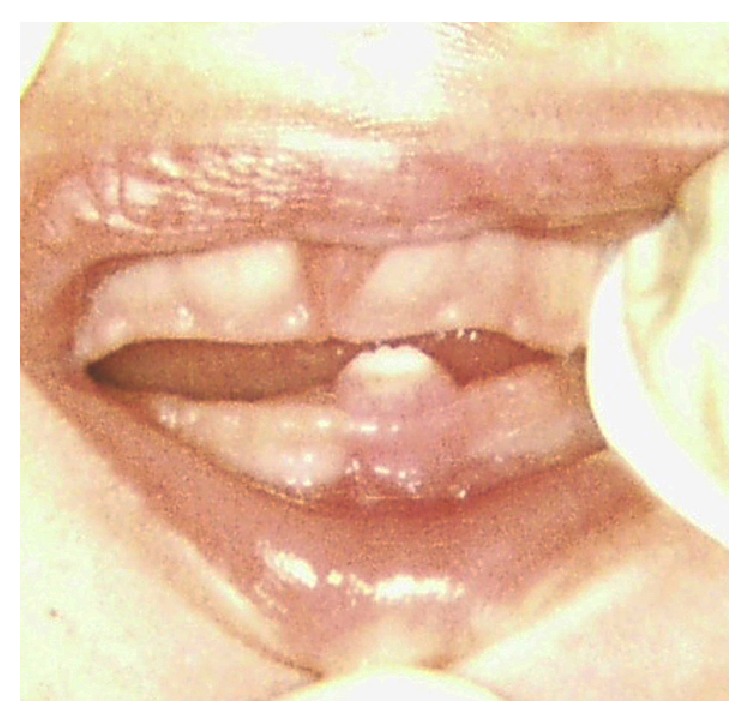
A 5-day-old female infant with partially erupting natal tooth in the anterior mandibular area, exhibiting grade II mobility.

**Figure 2:**
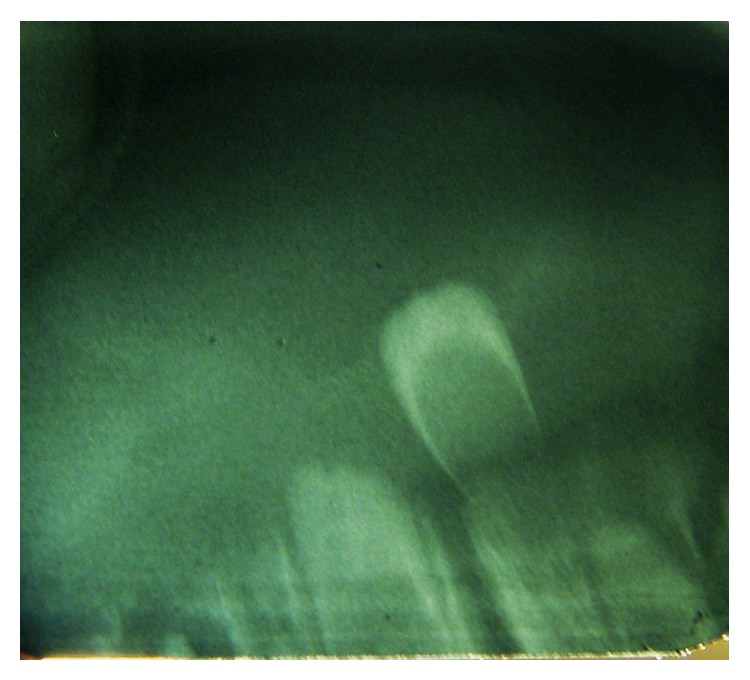
Periapical radiograph showing the natal tooth in the mandibular anterior area.

A five-day-old female infant was referred to the pediatric dental clinic, her mother was concerned about the presence of a tooth in the lower jaw since birth, and she complained of soreness during breastfeeding her child. Medical history was noncontributory. Extraoral examination showed a symmetrical face with no lymphadenopathy. Intraoral examination revealed a crown of a tooth in the mandibular anterior region, small in size, whitish opaque in color (Figure 1), and exhibiting grade II mobility. The lips, gingivae palate, tongue, floor of the mouth, and buccal mucosa were clinically normal in appearance and there was no ulceration on the ventral surface of the tongue. A diagnosis of natal tooth was made based on the clinical presentation and confirmed by a periapical radiograph (Figure 2).

After discussing the treatment options with the mother, it was decided that extraction was the best treatment since the mother was very concerned about the soreness of her breast and she felt she could not continue breastfeeding her child. Before extracting the natal tooth, a pediatrician was consulted and recommended that vitamin K (0.5–1.0 mg) to be given intramuscularly prior to the extraction to prevent potential hemorrhage. The natal tooth was then extracted under topical anesthesia and gentle curettage was performed to the extraction socket. The procedure was well tolerated by the infant. The extracted tooth had a crown but was lacking a root. The patient was reevaluated five days after extraction and at three months.

## 3. Discussion

The etiology of natal and neonatal teeth remains undetermined; however it was suggested to be related to various factors, including superficial position of the tooth germ, increased eruption rate due to pyretic incidents, hormonal stimulation, developmental abnormalities, syndromes, heredity, and osteoblastic activity within the germ zone related to the remodeling phenomenon [1, 7, 8, 16–24, 30, 31].

Natal and neonatal teeth could be either conical or of normal shape and size. They usually have an opaque yellow-brownish color. The dimensions of the crowns of these teeth are smaller compared to primary teeth that have erupted normally [1, 8, 23, 24].

Clinically, natal and neonatal teeth can be classified according to their degree of maturity: (1) a mature natal or neonatal tooth is nearly or fully developed and has moderately good prognosis and (2) an immature natal or neonatal tooth is incomplete or having a substandard structure with a poor prognosis [8, 32].

Hebling et al. [33] suggested another clinical classification in their case report according to tooth morphology during eruption into the oral cavity: (1) shell-shaped crown that is poorly fixed to the alveolus by gingival tissue with root absence, (2) solid crown that is poorly fixed to the alveolus by gingival tissue with little or no root, (3) eruption of the incisal margin of the crown through the gingival tissues, and (4) gingival edema with palpable but unerupted tooth.

The enamel in natal and neonatal teeth is normal for the age of the children; however, once the teeth erupt prematurely, the uncalcified enamel matrix wears off due to incomplete mineralization leading to teeth becoming yellow-brown in color and continuous breakdown of enamel [6]. Furthermore, the increased mobility leads to dentin and cementum cervical changes and possible ensuing of Hertwig's sheath degeneration preventing root formation [34]. Several histological findings have demonstrated that, albeit normal structure of natal and neonatal teeth enamel, the mineralization process of enamel is interrupted by early eruption. Hence, the enamel is described as hypomineralized or dysplastic and is prone to discoloration and wear [30, 35–38].

Histological data on natal and neonatal teeth have also found that varying degrees of hypoplastic enamel cover the crowns of these teeth. The enamel thickness for natal teeth is 300 mm and for neonatal teeth it is 135 mm, whereas in normal primary teeth the enamel layer is between 1000 and 1200 mm [30]. The dentinal area did not reveal any significant differences compared to normal primary teeth; however some SEM studies of these teeth have shown large interglobular spaces with abnormal cell inclusions.

The correct diagnosis of natal and neonatal teeth is important so as to determine if these teeth are supernumerary or normal dentition. Bohn nodules and dental lamina cyst are additional oral manifestations that may be confused with these dental conditions; but they can be differentiated by radiographic examination.

Several factors should be considered before a treatment plan is decided: (1) degree of mobility and implantation, (2) convenience during suckling, (3) interference with breastfeeding, and (4) if the tooth is supernumerary or is part of the normal dentition.

If these erupted teeth are diagnosed as part of the normal dentition, maintenance in the mouth is considered the primary treatment option except if they become a source of injury to the baby. If they are implanted well, these teeth should be left in the arch and only removed when they interfere with feeding or when they are extremely mobile with a risk of aspiration. Indications for removal include risk of dislocation, subsequent aspiration, and traumatic injury to the baby's tongue and/or the maternal breast [29].

According to some investigators, the detection of Riga-Fede disease is an indication for natal/neonatal tooth removal; however, others do not recommend removal since an acute incisal margin can be relieved by smoothing [24]. Tomizawa et al. [39] reported that the treatment of Riga-Fede disease by layering the incisal edge with any photopolymerizable resin, which is facilitated in rapid healing of the ulcers. Having said that, most of these teeth exhibit evidence of hypomineralization and therefore limited surface area of enamel is available for resin bonding. Given these factors combined with the difficulties adequate bonding procedure from access to proper moisture control and then enamel surface etching renders resin retention uncertain. In addition, if the restoration fails, there is a risk that the composite resin could also be swallowed.

Natal/neonatal teeth that show mobility of more than 1 mm are indicated for extraction; this is due to the probability of aspirating or ingesting natal teeth. Another reason for the removal of the natal/neonatal tooth is to alleviate feeding difficulties or complications like Riga- Fede disease. If extraction is the treatment of choice, it can be deferred till the child is 10 days of age or more and has appropriate blood levels of vitamin K. This ten-day waiting period is to allow the normal flora of the intestine to become established to produce vitamin K, an essential factor for prothrombin production in the liver [1, 8, 37]. Since parenteral vitamin K prevents a life threatening haemorrhagic disease of the newborn, the American Academy of Pediatrics recommends that all newborns be given a single intramuscular dose of 0.5 to 1 mg of vitamin K [40]. If it is not possible to delay the extraction, a consultation with the pediatrician should be initiated, so they can assess if there is a need to administer vitamin K, if the newborn did not receive vitamin K immediately after birth.

Once extraction is performed, it is essential to remove the underlying dental papilla and Hertwig's epithelial root sheath during the extraction of natal tooth/teeth to prevent the development of root structure that could continue if these structures are left* in situ.*

## 4. Conclusions

Natal and neonatal teeth are rare occurrences in the oral cavity and proper evaluation and diagnosis are crucial to provide the best treatment option. Pediatricians are usually the first to detect these teeth and early consultation with the dentist can prevent complications. The decision to maintain or remove these teeth should be assessed in each case independently. Radiographic examination is an essential diagnostic tool. Thus far, no studies confirmed the cause and effect relationship with any of the proposed theories so far. The etiology of natal and neonatal teeth still required further investigations.
